# Are Panoramic Radiographs Reliable to Diagnose Mild Alveolar Bone Resorption?

**DOI:** 10.5402/2011/363578

**Published:** 2011-05-04

**Authors:** Larissa Semenoff, Tereza Aparecida Delle Semenoff, Fabio Luiz Miranda Pedro, Evaristo Ricci Volpato, Maria Aparecida de Andrade Moreira Machado, Álvaro Henrique Borges, Alex Semenoff-Segundo

**Affiliations:** ^1^Department of Radiology, Especialista em Radiologia, UNIDERP, Rua Ceará 333, 79003-010 Campo Grande, MS, Brazil; ^2^Department of Endodontics, Dental School, University of Cuiabá, Avenida Beira Rio 3100, 78065 900 Cuiabá, MT, Brazil; ^3^Department of Pediatric Dentistry, Dental School, University of Cuiabá, Avenida Beira Rio 3100, 78065 900 Cuiabá, MT, Brazil; ^4^Department of Pediatric Dentistry, Orthodontics and Community Health, Bauru School of Dentistry, University of São Paulo, Al. Octávio Pinheiro Brisola, 9-75, 17012-901 Bauru, SP, Brazil; ^5^Discipline of Hospital Dentistry, University of Cuiabá, Rua Professora Azélia de Mamoré de Melo, no. 318, Ap. 63, 78005-700 Cuiabá, MT, Brazil

## Abstract

It is extremely important to assess variations between the most used radiographs in dental practice, since minimum distortion on obtained images may change diagnosis, treatment plan, and prognosis for the patient. For this, the distance between the enamel-cementum junction and the alveolar bone crest was measured on conventional and digitized periapical, bitewing, and panoramic radiographs and compared among them. From a total of 1484 records, 39 sets of radiographs that fulfilled the inclusion criteria of the study sample were selected. The measurements were grouped according to the intensity of bone loss. Statistically significant difference was found in the averages of the measurements assessed in radiographs with absence of bone loss between conventional panoramic and periapical radiographs, between digitized panoramic and periapical radiographs and between digitized bitewing and panoramic radiographs. By analyzing the results of this work and considering the research protocol used, one can conclude that small losses in height of alveolar bone crest observed in panoramic radiographs should be cautiously evaluated, as they may be overestimated.

## 1. Introduction

Radiographic examination is part of routine dental treatment, with periapical, bitewing, and panoramic radiographs being the most commonly used. These are conventional radiographs, which are capable of diagnosing fractures, pathologic lesions and development abnormalities. Such radiographs might be employed in epidemiologic surveys to large populations, being their cost-benefit relation for the patient in aiding the definition of a diagnosis and prognosis very satisfactory [1].

Periapical radiographs are indicated to assess the width of periodontal ligament, bone trabecular pattern and density, size of the root trunk, root divergences, and presence of lateral or periapical lesions and combined lesions (endodontic-periodontal) [2]. Bitewings by their turn present good reading precision at the alveolar crest and cementum-enamel junction areas, and thus, provide reliable information in comparison to clinical findings [3]. Intraoral radiographs are also important for the diagnosis and monitoring of marginal bone levels [4].

The panoramic radiograph is excellent for visualization of general structures of the face. This radiograph is also performed when difficulties in performing Intraoral radiographs are experienced. However, frequent distortions to mesial-distal measurements are observed, limiting a more precise evaluation mainly to the anterior segment and maxillary molars areas [4].

New diagnostic image exams have been recently introduced to dental practice, such as digital radiographs and computed tomography. More modern technologies allow three-dimensional visualization of images of the maxillofacial complex, leading to a more precise planning and execution of the dental treatment with better predictability [5]. However, the high cost of equipments and consequently the exams is still a very relevant disadvantage to be considered, which frequently reduces their employment [6]. Another obstacle to be overcome is related to the lack of utilization of digital reading systems by great part of dentists. 

Thus, it is very important to evaluate existent variations among the most commonly employed radiographic examinations used at dental settings, once minimum distortions may change diagnosis, treatment plan, and prognosis for patients.

## 2. Material and Methods

**Figure 1:**
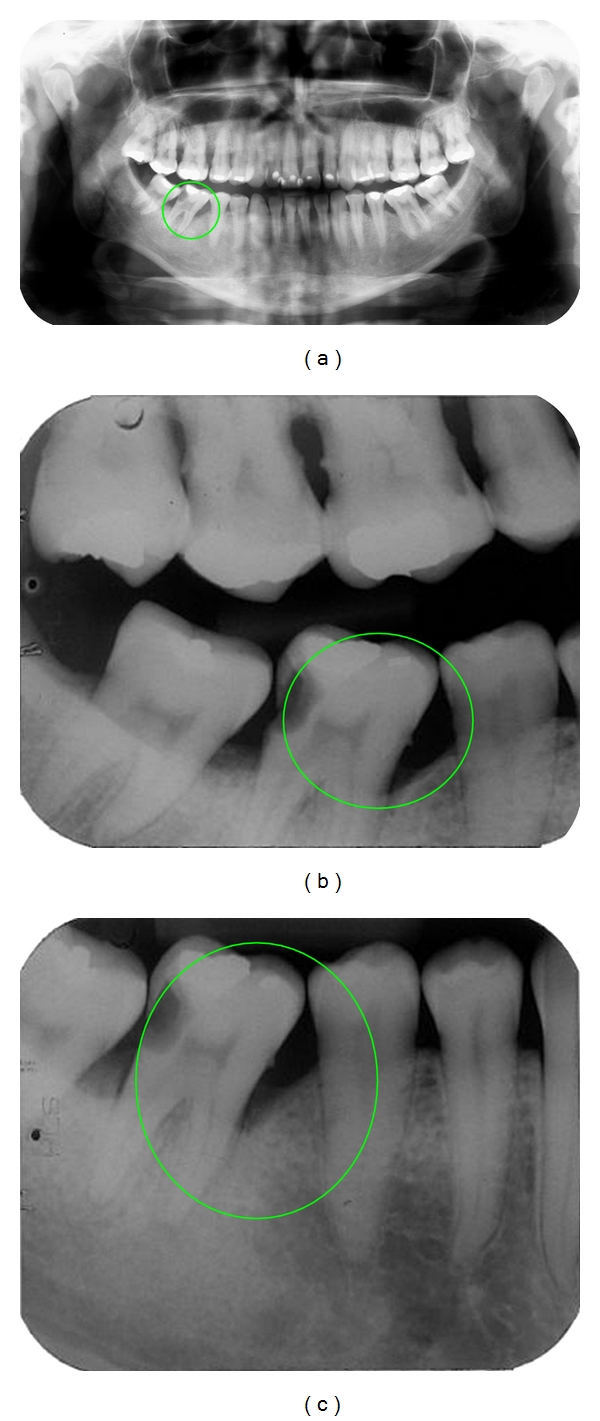
Illustrative figures of the images manually measured: panoramic (a), bitewing (b) and periapical radiographs (c).

The study was approved by the IRB of the Public Health School of the Mato Grosso State Department (Protocol no. 367/08). One thousand four hundred and eighty four charts from a private practice located in Jaciara, Mato Grosso were selected. Seventy four of those presented panoramic (Figure 1(a)), bitewing (Figure 1(b)), and periapical radiographs (Figure 1(c)).

Inclusion criteria for the patients were presence of a set of good quality radiographs, availability to go through the digitization process, well-defined images at the anatomic neck (cementum-enamel junction) and alveolar bone crest at the level of first and second mandibular molars, and presence of at least one adjacent tooth and satisfactory positioning of the tooth in the dental arch.

Exclusion criteria were radiographs performed in different dates, images that did not allow adequate evaluation, no automated radiographic development employed, and presence of accentuated angulations of mandibular molars in relation to adjacent teeth.

Thirty-nine radiograph sets followed the inclusion criteria for the study and were digitized using a photographic scanner (HP Scanjet G4000 series, Barueri, SP, Brazil), attached to a laptop (Toshiba-Satellite A-10-S129, Hong Kong, China). The images were manipulated using a dental radiology software (Radiomemory, Belo Horizonte, MG, Brazil). Reddy in 1997 reported the advantages of image digitizing in Dentistry, due to precision of performed measurements and due to the possibility of using softwares to treat the images. 

In order assure the standardization of analyses, all the measurements were performed by a single evaluator, being previously calibrated for the study. The agreement between the performed evaluations was measured by the student paired* t*-test, being 0.986 with a standard error of 0.08 mm. The measurements of the conventional radiographs were performed with aid of a table negatoscope and a millimeter ruler, while the digitized radiographs were evaluated through a specific software for radiograph measurements, Radio Memory. Every measurement was performed in a dark environment aiming better visualization of images. 

The distance between the anatomic tooth neck of the second mandibular molar on the mesial portion and the bone crest of the side presenting the greatest apical bone loss in height (right or left) was the chosen measurement for comparison between the set of radiographs. 

The observed measurements for both conventional and digitized radiographs were classified according to the following categories: 0–2 mm (absence of bone loss), 3–5 mm (moderate bone loss), and ≥6 mm (advanced bone loss) and compared among them.

The observed variations were submitted to the analysis of variance with Bonferroni correction at a level of significance of 5%.

## 3. Results

The average of bone loss in millimeters was determined after the radiographs were distributed into groups according to the measured bone loss, followed by the standard deviation. These data are presented on Table 1.

In this table, differences were searched between the obtained average measurements within the conventional or digitized radiograph groups or between them. It was found that the average of measurements of the groups presenting moderate or advanced bone loss did not present any statistical significant difference within either the conventional or digitized radiograph groups. 

Among the radiographs presenting no bone loss, statistical significant differences were observed between the averages observed on the conventional periapical and panoramic radiographs (*P* = .03), between the measurements of the digitized periapical and panoramic radiographs (*P* = .01), and between the digitized bitewing and panoramic radiographs (*P* = .02).

There were no statistical significant differences on the mean measurements between conventional and digitized radiographs according to the type of radiograph.

## 4. Discussion

The radiographic examination, besides its limitations, is a fundamental tool to diagnosis and to follow up the evolution of performed treatments, especially for patients under periodontal therapy [1, 4, 7]. Periapical and bitewing radiographs are the most indicated to identify alterations on sustaining periodontal tissues, mainly for alterations involving bone loss [3, 8, 9]. However, some factors are related to the greater utilization of panoramic radiographs, such as increased number of radiology centers resulting in a more accessible procedure, decrease in price of panoramic radiographs, exam convenience, as the film is positioned outside patients' mouth, and professionals' intention to reduce X-ray exposure, as a single exposure for panoramic radiographs during a check-up substitutes multiples exposures required for periapical and bitewing radiographs [2, 9, 10].

In a study performed by Papapanou and Wennstrom [10], in 1989, clinical measurements were compared to radiographic evaluations obtained from periapical and bitewing radiographs. Greater discrepancies were found to patients presenting larger periodontal destruction. On the other hand, Li et al. [4] (2007) reported that the severity of marginal bone loss did not affect measurements precision when comparing clinical and radiographic measurements, regardless the intensity of the marginal bone loss. Another study following this same research focus [4] compared panoramic with periapical and bitewing radiographs in patients presenting aggressive and advanced periodontitis, and the results presented differently from the present study. This disparity in results certainly occurred as the included patients presented large bone loss. Another point for discussion and disagreement is related to the fact that the evaluation criteria for differences among the groups also differ. It is also important to point out that in this study the different results were observed for the small bone loss group, which highlights proper care required when analyzing early bone loss in patients possibly presenting periodontitis.

The present study observed statistical significant differences only in cases of bone loss lower than 2 mm, contradicting previously reported studies [2, 5, 8]. When comparing the conventional radiographs, it was observed that the average bone loss assessed on the periapical radiographs was 32% lower than the data obtained from the panoramic radiographs.

The analysis of the digitized radiographs followed the same pattern presented on the conventional ones. However, during the digitized assessment, bitewings also presented inferior bone loss averages when compared to the panoramic images. The precision of measurements in digitized radiographs probably decreased the variation of central tendency measurements (standard deviation and standard error), resulting in statistical differences between them. The results presented might have had some influences by the sample size; however, the inclusion criteria for case selection provide consistency to the results. Other researches compared measurements of marginal alveolar bone loss using digitized radiographs incorporating color or not [4] and digitized radiographs with original or inverted colors [7], and no statistical significant difference was reported for both cases.

The assessment of mandibular molars was the choice in the present study, as the cementum-enamel junction and the alveolar bone crest are easily assessed and consequently the distance between them [7]. It is highlighted that the sample was composed by teeth positioned on an adequate plane in relation to adjacent teeth. 

In relation to the radiographs, they were all taken at the same day for each patient; film holders were used and they were developed using automatic developers at only three radiologic centers. Although these steps are important for the measurements accuracy [1], it is known that nonstandardized periapical and bitewing radiographs might not change the research measurements [11].

The results of this study presented great clinical relevance in demonstrating that small losses at bone level observed on panoramic radiographs should be cautiously interpreted once they might be overestimated. The same losses were significantly lower when observed on bitewing and periapical radiographs, which present good similarity with clinical inspection [4, 5, 10].

## 5. Conclusion

By the analyses of the data of the present study and considering the employed research protocol, it can be concluded that small loss in height of alveolar bone crest observed on panoramic radiographs might be carefully considered, once they can be overestimated.

## Figures and Tables

**Table 1:** Distribution of mean measured distances according to the type of radiograph.

Type of radiograph	Bone loss (in millimeters)		
	Absent	Moderate	Advanced
Conventional periapical	1.70 ± 0.48*	3.78 ± 0.85	7.10 ± 1.28
Digitized periapical	1.70 ± 0.45^*∂*^	3.76 ± 0.84	7.20 ± 1.50
Conventional bitewing	1.90 ± 0.56	3.57 ± 0.96	6.60 ± 1.65
Digitized bitewing	1.78 ± 0.37^†^	3.64 ± 1.02	6.60 ± 1.75
Conventional panoramic	2.50 ± 0.84*	3.89 ± 1.28	8.00 ± 2.30
Digitized panoramic	2.59 ± 0.89^*∂*†^	3.98 ± 1.18	8.00 ± 2.30

*Significant difference between conventional periapical and panoramic radiographs (*P* = .03).

^*∂*^Significant difference between digitized periapical and panoramic radiographs (*P* = .01).

^†^Significant difference between digitized bitewing and panoramic radiographs (*P* = .02).
